# Reconstruction of a Full-Thickness Right Nasal Ala Defect After Basal Cell Carcinoma Excision Using a Nasolabial Flap: A Single-Stage and Functional Approach

**DOI:** 10.7759/cureus.90602

**Published:** 2025-08-20

**Authors:** Guillermo Sergio Dorantes-Millan, Cristina Lizbeth Puntos-Guizar, Roberto Rodriguez-Ramirez, Uriel Guillen-Morales, Luis Eduardo Escobar-Rios

**Affiliations:** 1 Surgery, Hospital General Dr. Fernando Quiroz Gutierrez, Mexico City, MEX; 2 Dermatology, Centro Medico Nacional 20 De Noviembre, Mexico City, MEX; 3 General Surgery, Hospital General Dr. Fernando Quiroz Gutierrez, Mexico City, MEX

**Keywords:** basal cell carcinoma, facial reconstruction, flap, nasolabial flap, plastic and reconstructive surgery

## Abstract

Basal cell carcinoma (BCC) is the most prevalent form of non-melanoma skin cancer, involving exposed areas such as the nose. Its location in this anatomically and aesthetically sensitive region poses significant diagnostic and therapeutic challenges, requiring a balance between complete tumor removal and preservation of nasal function and appearance. We present the case of a 64-year-old female cleaning worker with an eight-year history of a gradually enlarging lesion on the right nasal ala, which was diagnosed as a micronodular BCC following biopsy. The micronodular BCC is associated with more aggressive behavior and a higher risk of recurrence. This is why wider surgical margins and careful management are necessary in this type of disease to ensure complete tumor eradication. The patient underwent surgical excision with a 5 mm margin, including the full thickness of the nasal ala. Immediate reconstruction was successfully carried out using a nasolabial flap, a versatile local flap renowned for its excellent match in color, texture, and thickness to the nasal skin, along with its reliable vascular supply and minimal donor site morbidity.

Histopathological examination showed free-margin, complete tumor resection and no lymphovascular or perineural invasion. The patient’s postoperative evolution was uneventful, with no flap-related complications. At the two-month follow-up, the surgical site had healed with a scar that was discreetly camouflaged within the natural nasolabial fold, preserving nasal contour and facial symmetry. This case highlights the functional and cosmetic benefits of the nasolabial flap for full-thickness nasal ala defect reconstruction, as timely reconstruction of the defect using local flaps not only enhances the functional and cosmetic outcomes but also results in better patient quality of life.

## Introduction

Basal cell carcinoma (BCC) is the most common cutaneous neoplasm, accounting for up to 80% of non-melanoma skin cancers. Its frequent occurrence in photoexposed areas, such as the nose, represents both a diagnostic and therapeutic challenge due to its locally invasive behavior and the aesthetic and functional importance of this region [1]. Surgical excision with adequate margins remains the standard treatment, especially when Mohs micrographic surgery is not available. Margins ranging from 3 to 7 mm have been demonstrated to achieve complete tumor removal in most cases [1].

In high-risk locations like the nose, Mohs micrographic surgery is considered the treatment of choice as it allows for layer-by-layer excision with immediate histopathological assessment, optimizing oncological outcomes while preserving healthy tissue [2]. Nevertheless, due to limited availability, many centers perform conventional excision followed by immediate reconstruction.

The use of local facial flaps in nasal reconstruction has evolved significantly, with a growing body of literature supporting tunneled island flaps, including nasolabial flaps, as a safe and effective option for single-stage nasal reconstruction [3]. The nasolabial or melolabial flap has gained widespread acceptance as a versatile and effective option, particularly useful for reconstructing the nasal ala, columella, lateral nasal wall, and even intraoral areas. This flap follows a random vascular pattern, with blood supply primarily from branches of the angular, superior labial, and dorsal nasal arteries [4].

One of the main advantages of the nasolabial flap is its excellent compatibility in color, thickness, and texture with the skin of the nasal region, which allows for superior aesthetic integration. In addition, its design and rotation are technically accessible, making it a reliable option for both small and full-thickness defects of the nasal ala. Its versatility allows reconstructions to be performed in a single surgical stage, with low complication rates, good tissue perfusion, and discrete scars that are camouflaged in the nasolabial fold [5,6]. These characteristics position it as one of the preferred flaps for restoring nasal contour without compromising respiratory function or facial symmetry.

The nose is one of the areas most commonly affected by basal cell and squamous cell carcinomas of the cephalic region, and its reconstruction depends on the size and location of the defect. Options include skin grafts, local flaps, or free flaps [6]. In particular, nasolabial flaps present an excellent adaptation for restoring the shape of the nasal ala, as their color and texture closely resemble those of the alar skin, allowing for harmonious aesthetic integration [5].

In addition, different types of nasolabial flaps have been shown to be effective in reconstructing full-thickness defects of the nasal ala in a single surgical procedure, providing good functional outcomes without the need for complex multilayer reconstruction techniques [6]. These methods follow the facial subunit principle, which focuses on hiding scars within the natural folds and creases of the face to achieve the best possible aesthetic result [6].

## Case presentation

**Figure 1:**
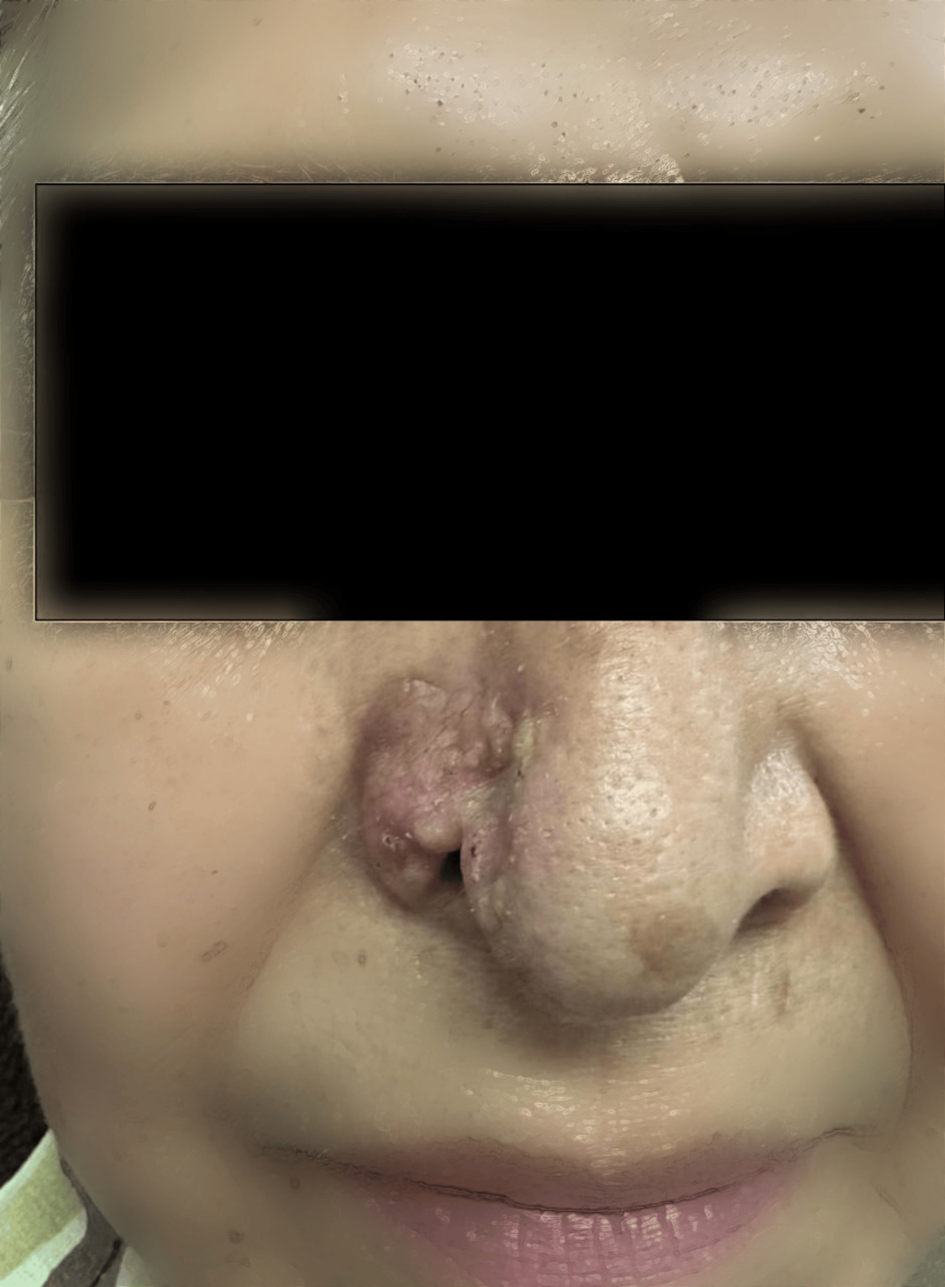
Preoperative view showing the full-thickness defect of the right nasal ala. The ulcerated basal cell carcinoma lesion is visible.

A 64-year-old female cleaning worker with no significant family history of cancer presented with an eight-year history of a slowly progressing lesion on the right nasal ala. The lesion initially measured approximately 0.5 x 0.5 cm, erythematous in appearance, and over time exhibited gradual enlargement with the development of a central ulcer during the last year. She was evaluated by the dermatology department, where a biopsy was performed and reported a solid micronodular BCC, infiltrating and ulcerated. Physical examination showed a verrucous tumor with a bleeding base of approximately 2 x 1.5 cm, covering the right nasal ala with destruction of the central border and peripheral telangiectasias (Figure 1). 

**Figure 2:**
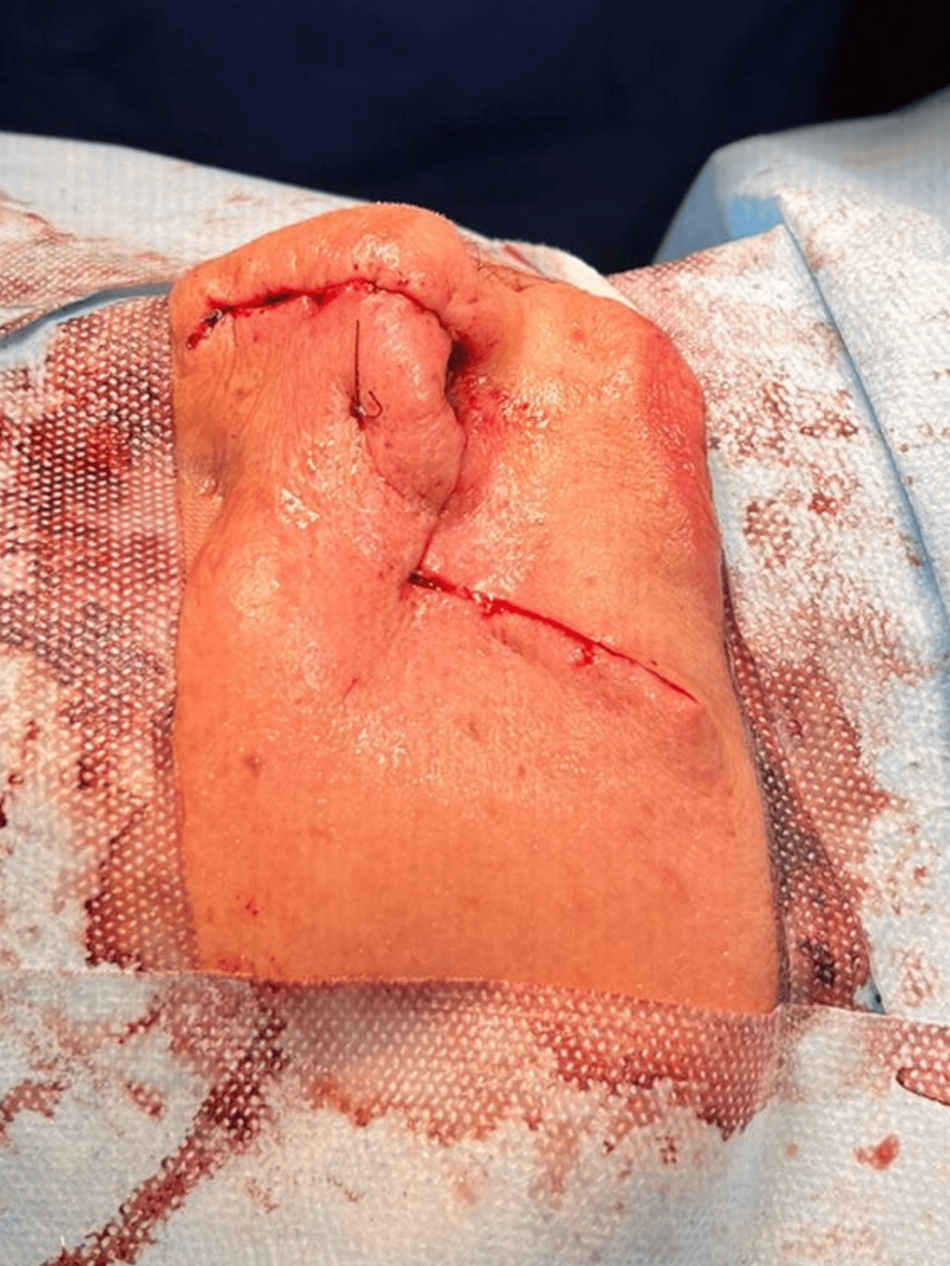
Intraoperative view following immediate reconstruction of a full-thickness right nasal ala defect using a nasolabial flap. The image shows the nasolabial flap rotated and inserted into the defect.

No cervical adenopathies were palpable. Based on the patient’s clinical history, physical examination, and histopathological report, a preoperative protocol was initiated. Preoperative evaluations (Goldman II, ASA II) indicated no contraindications for surgery. Following surgical marking, the nasal pyramid and right nasal ala were prepared with antiseptic and aseptic techniques, and sterile surgical drapes were applied. Local infiltration with 2% lidocaine and epinephrine was administered in the nasal region. A circumferential incision with a 5 mm margin was made around the lesion, encompassing the full thickness of the nasal ala. The tumor was excised with adequate margins. Immediate reconstruction was performed using a nasolabial flap, elevating and rotating a flap approximately 6 x 4 cm in size to cover the defect (Figure 2). 

The flap was sutured to the subcutaneous tissue with 3-0 Vicryl, and the skin was closed with 4-0 nylon sutures. Hemostasis and flap viability were carefully assessed, confirming adequate perfusion and coloration. The surgery was concluded with the application of sterile dressings and nasal packing.

**Figure 3:**
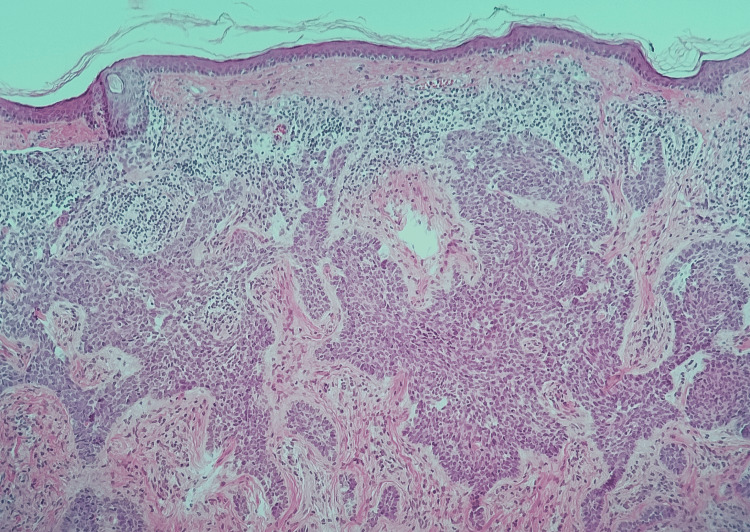
Low-magnification image (10X) showing thin skin with scarce stratum corneum and abundant chronic inflammatory infiltrate in relation to nests of basaloid cells invading superficial and deep dermis.

The histopathological analysis confirmed an ulcerated micronodular BCC measuring 2 x 1.5 cm, with clear surgical margins and no evidence of lymphovascular invasion or perineural infiltration (Figure 3).

**Figure 4:**
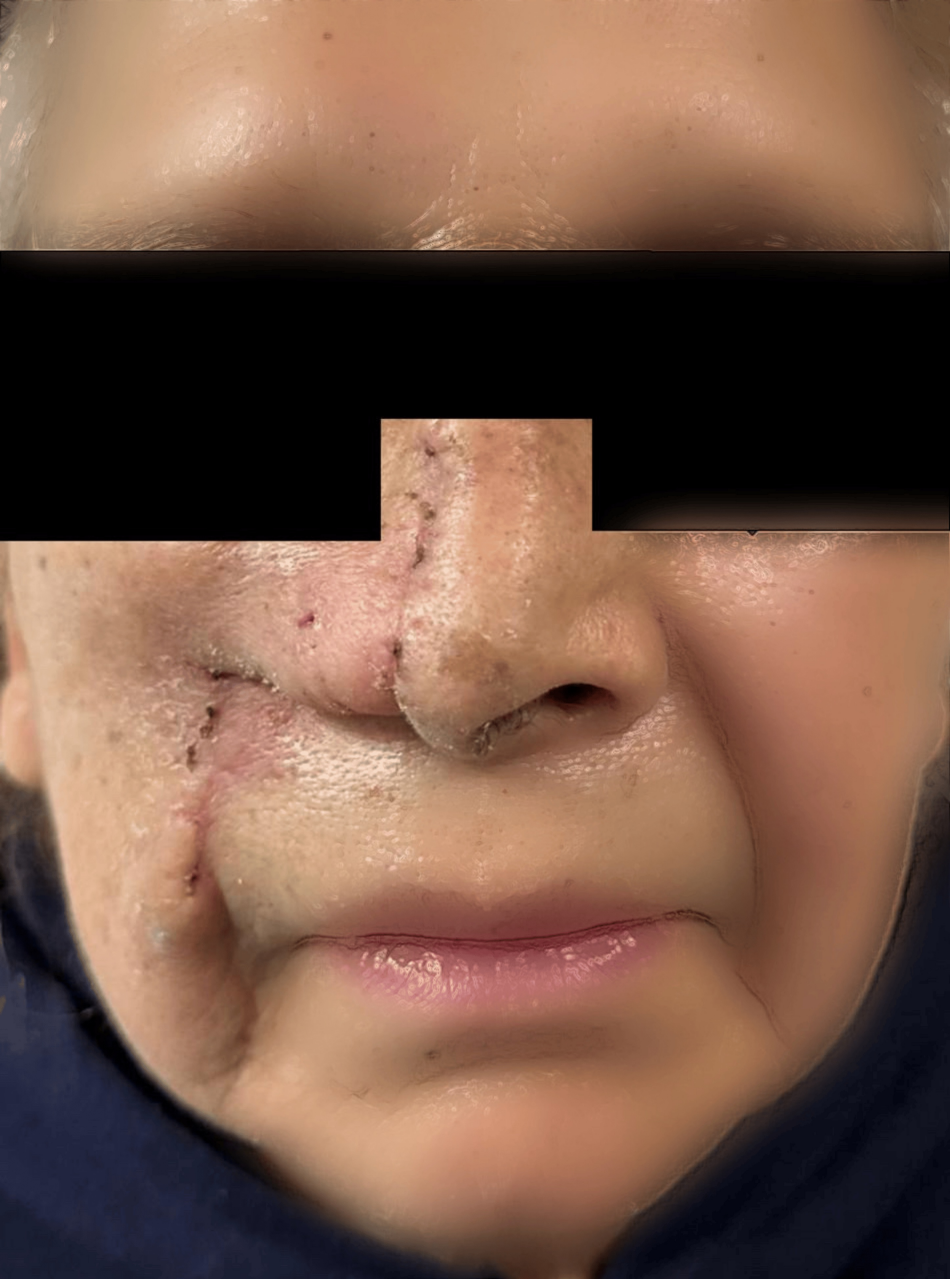
Patient in the postoperative period. A scar line is observed extending from the nasofacial region toward the nasolabial fold, corresponding to the design and inset of the nasolabial flap.

The patient demonstrated a favorable postoperative course, without any early complications related to the flap. She was discharged on postoperative day three with scheduled follow-up in the oncology department. At the two-month follow-up, the surgical site showed a well-healed scar measuring approximately 6 cm in length and 2 mm in width on the right side, with the patient maintaining good clinical condition (Figure 4).

## Discussion

Surgical treatment of nasal BCC should be comprehensive, considering not only complete oncologic eradication, but also functional and esthetic restoration of the affected area. Immediate reconstruction with local flaps, such as the nasolabial flap, has proven to be safe and efficient. When compared to other reconstructive options, such as the paramedian forehead flap, the nasolabial flap offers several key advantages: lower donor site morbidity, superior integration with the native facial contour, and a shorter recovery period. Recent studies have shown that patients who undergo reconstruction with nasolabial flaps report higher levels of aesthetic satisfaction, reduced scar contracture, and significantly faster postoperative recovery [4].

In selected cases, even full-thickness defects of the nasolabial ala can be effectively reconstructed using the turnover-type nasolabial flap technique. This method offers a reliable single-stage alternative that eliminates the need for complex, multistage procedures. Schonauer and colleagues (2024) shared their experience with this technique, showing that it can be performed successfully without the need for cartilage or mucosal grafts, and with no major complications [5].

While the nasal ala presents a unique reconstructive challenge due to its complex anatomy and central role in facial aesthetics, the nasolabial flap has established itself as a reliable solution. Although the paramedian forehead flap has long been considered the “workhorse” for large nasal defects, thanks to its ample, pliable tissue, it typically requires at least two surgical times and often requires cartilage harvesting, commonly from the auricular concha. This may result in visible donor site scarring, which can be aesthetically unsatisfactory for some patients. By contrast, the nasolabial flap provides a valuable alternative for full-thickness nasal ala reconstruction. Its inherent pliability, robust vascularity, appropriate thickness, and three-dimensional adaptability allow it to effectively replicate the anatomical structure of the nasal ala without requiring cartilaginous support [5-7].

Recent analyses on reconstruction for BCC of the face emphasize the importance of the facial subunit principle. This approach allows surgical scars to blend naturally within the contours and form of the nose, improving the outcome and ensuring full harmony with the rest of the nasal architecture [6].

Ultimately, while the paramedian forehead flap remains a key option for extensive nasal defects, current evidence indicates that the nasolabial flap provides excellent functional and aesthetic outcomes in mild to moderate cases. It is associated with fewer surgical stages, reduced morbidity, and improved contour integration and patient satisfaction. A recent systematic review confirmed that the nasolabial flap demonstrates complication rates comparable to or even lower than those of the forehead flap, with consistently high levels of aesthetic and functional satisfaction in appropriately selected nasal reconstructions [7].

## Conclusions

The increasing presence of oncologic lesions in the facial region requiring extensive resections with a history of reported pathology of non-infiltrative micronodular basocellular carcinoma continues to represent a major reconstructive challenge. This is especially true in cases involving large defects, such as full-thickness defects of the nasal ala following excision of BCC, where treatment must balance adequate oncologic resection with preservation of facial function and aesthetics. The nasolabial flap stands out as a reliable, versatile, and low-risk option that allows an anatomical restoration in a single surgical time, with good skin integration and minimal donor site morbidity. In the case presented, we obtained good results not only with tumor-free surgical margins but also with adequate preservation of nasal function and a satisfactory outcome for the patient. Based on current evidence, the nasolabial flap should be considered as a first-line option for mild to moderate nasal defects, especially when seeking to avoid more complex or multistage procedures without compromising the quality of the final result and functionality. 

Given the higher recurrence risk associated with the micronodular basocellular carcinoma, even after complete excision, microscopic tumor cells may remain, favoring local recurrence. Long-term patient monitoring is recommended. Recognizing this subtype helps guide wider surgical margins, careful management, and close follow-up. Further studies are needed to validate these findings, as conclusions are limited by the presentation of a single case.
